# Antioxidant and Anti-Inflammatory Effects of Curcumin Nanoparticles on Drug-Induced Acute Myocardial Infarction in Diabetic Rats

**DOI:** 10.3390/antiox8100504

**Published:** 2019-10-22

**Authors:** Paul-Mihai Boarescu, Ioana Boarescu, Ioana Corina Bocșan, Dan Gheban, Adriana Elena Bulboacă, Cristina Nicula, Raluca Maria Pop, Ruxandra-Mioara Râjnoveanu, Sorana D. Bolboacă

**Affiliations:** 1 Department of Pathophysiology, Iuliu Haţieganu University of Medicine and Pharmacy Cluj-Napoca, Victor Babeş Street, No. 2–4, 400012 Cluj-Napoca, Romania; boarescu.paul@umfcluj.ro; 2 Department of Medical Informatics and Biostatistics, Iuliu Haţieganu University of Medicine and Pharmacy Cluj-Napoca, Louis Pasteur Street, No. 6, 400349 Cluj-Napoca, Romania; sbolboaca@umfcluj.ro; 3 Department of Neurology, County Clinical Emergency Hospital of Cluj-Napoca, Victor Babeș Street, No. 43, 400012 Cluj-Napoca, Romania; ioana.boarescu@gmail.com; 4 Department of Pharmacology, Toxicology and Clinical Pharmacology, Iuliu Haţieganu University of Medicine and Pharmacy Cluj-Napoca, Gheorghe Marinescu Street, No. 23, 400337 Cluj-Napoca, Romania; corinabocsan@yahoo.com; 5 Department of Pathological Anatomy, Iuliu Haţieganu University of Medicine and Pharmacy Cluj-Napoca, Clinicilor Street, No. 3–5, 400006 Cluj-Napoca, Romania; dgheban@gmail.com; 6 Department of Ophthalmology, Iuliu Haţieganu University of Medicine and Pharmacy Cluj-Napoca, Clinicilor Street, No. 3–5, 400006 Cluj-Napoca, Romania; 7 Department of Pneumology, Iuliu Haţieganu University of Medicine and Pharmacy Cluj-Napoca, B.P. Hasdeu Street, No. 6, 400371 Cluj-Napoca, Romania; andra_redro@yahoo.com

**Keywords:** streptozotocin (STZ), isoproterenol (ISO), oxidative stress, cytokines, cardio-protection

## Abstract

We have investigated the cardio-protective effects of pretreatment with curcumin nanoparticles (CUN) compared to conventional curcumin (CUS) on the changes in oxidative stress parameters and inflammatory cytokine levels during induced acute myocardial infarction (AMI) in rats with diabetes mellitus (DM). DM was induced with streptozotocin, and AMI with isoproterenol. Eight groups of seven Wister Bratislava rats were included in the study. The N-C was the normal control group, AMI-C was the group with AMI, DM-C was the group with DM, and DM-AMI-C was the group with DM and AMI. All four groups received saline solution orally during the whole experiment. S-DM-CUS-AMI and S-DM-CUN-AMI groups received saline for seven days prior to DM induction and continued with CUS (200 mg/kg bw, bw = body weight) for S-DM-CUS-AMI and CUN for S-DM-CUN-AMI (200 mg/kg bw) for 15 days before AMI induction. The CUS-DM-CUS-AMI group received CUS (200 mg/kg bw), while the CUN-DM-CUN-AMI received CUN (200 mg/kg bw) for seven days prior to DM induction, and both groups continued with administration in the same doses for 15 days before AMI induction. CUS and CUN prevented elevation of creatine kinase, creatine kinase-MB, lactate dehydrogenase in all groups, with better results in the CUN (S-DM-CUN-AMI and CUN-DM-CUN-AMI groups). CUS and CUN significantly reduced serum levels of oxidative stress markers (malondialdehyde, the indirect assessment of nitric oxide synthesis, and total oxidative status) and enhanced antioxidative markers (total antioxidative capacity and thiols, up to 2.5 times). All groups that received CUS or CUN showed significantly lower serum levels of tumor necrosis factor-alpha, interleukin-6, and interleukin-1β. The best antioxidative and anti-inflammatory effects were obtained for the group that received CUN before DM induction (CUN-DM-CUN-AMI group). Pretreatment with CUN proved higher cardio-protective effects exerting an important antioxidative and anti-inflammatory impact in the case of AMI in DM.

## 1. Introduction

Diabetes mellitus (DM) is a chronic metabolic disorder defined by hyperglycemia as a result of defects in insulin secretion (type 1 DM), insulin action (type 2 DM), or both (type 2 DM) [1]. Chronic complications of DM, responsible for the elevated morbidity and mortality, can be divided into vascular (e.g., macrovascular-coronary artery disease, cerebrovascular disease, and peripheral vascular disease, and microvascular-neuropathy, retinopathy, and nephropathy) and nonvascular complications, and they may involve several different organs [2].

An important issue in the management of DM is the prevention of macrovascular complications such as stroke and acute coronary syndromes [3]. The acute coronary syndrome encloses a spectrum of conditions that include unstable angina pectoris, acute myocardial infarction (AMI), and, to some extent, sudden cardiac death. AMI, an important acute disease of the myocardial tissue, caused by an imbalance between low coronary blood delivery and high myocardial blood demand, is the leading cause of morbidity and mortality worldwide [4]. This imbalance leads to ischemia of myocardial tissue with degeneration of cardiomyocytes and eventually to irreversible cardiac injury and death [4]. The incidence of AMI among diabetic patients is almost 2 times higher than those without DM, as a result of cumulative cardiovascular risk factors that these patients are associated with, which are, namely, hyperglycemia, dyslipidemia, chronic inflammation, and oxidative stress [5]. In patients with DM, prolonged hyperglycemia was found to participate in the pathogenesis of AMI since it enhances oxidative stress in the heart tissue and overstates the associated damage by increasing membrane lipid peroxidation and inflammation [6].

Streptozotocin (STZ) [2-deoxy-2-(3-(methyl-3-nitrosoureido)-d-glucopyranose] is an antibiotic and one of the most effective diabetogenic chemicals, being widely used to induce type 1 DM in experimental models [7]. After intraperitoneal (ip) or intravenous (iv) administration, STZ, a glucose analog, enters pancreatic β cells via the Glut-2 transporter [8]. STZ has pancreatic β-cell-specific cytotoxic effects and induces deoxyribonucleic acid (DNA) alkylation and fragmentation [9]. Subsequently, the fragmented DNA activates reparative enzymes, leading to a depletion of adenosine triphosphate (ATP), increased oxidative stress, alterations in cellular metabolism, mitochondrial dysfunction, and eventually necrosis of β-cells, followed by β-cells loss and atrophy of the islets [9]. The destruction of β islets results in an absolute or relative deficit of insulin and hyperglycemia [10].

Isoproterenol (ISO) [1-(3,4-dihydroxyphenyl)-2-isopropylamino ethanol hydrochloride] is a synthetic catecholamine, and β-adrenergic receptor agonist, which, administered in high doses, causes myocardial infarction-like lesions [11]. After administration, ISO generates increased lipid peroxidation, antioxidant depletion, synthesis of inflammatory cytokines, intracellular Ca^2+^ overload, and apoptosis, leading eventually to myocardial necrosis [12]. The experimental model of AMI induction with ISO is a well-established model to study the beneficial effects of active compounds, including those with antioxidant and anti-inflammatory effects [13,14,15,16].

Curcumin is a natural polyphenol and essential curcuminoid derived from the rhizome of the medicinal plant *Curcuma longa*, reported to possess antioxidative, anti-inflammatory, anticarcinogenic, and cardiovascular protective effects [17,18,19]. Curcumin has been used in traditional Asian medicine to treat a variety of chronic diseases, including rheumatoid arthritis, psoriasis, vitiligo, diabetes mellitus, colon or pancreatic cancer, and cognitive dysfunctions [20,21].

Curcumin has received considerable attention for the management of DM and its complications because it was proved to be effective in reducing hyperglycemia and hyperlipidemia in rodent models, and it is also safe and relatively inexpensive [1]. Apart from the antioxidative and anti-inflammatory effects in AMI [14,15], curcumin was also observed to have cardio-protective effects, as it reduces necrosis and apoptosis of cardiomyocytes; it may also reduce complications associated with myocardial infarction [22].

The major limitation of curcumin usage after oral administration is the low bioavailability due to low aqueous solubility and gastrointestinal absorption, rapid metabolism, systemic elimination, and alkaline pH degradation [23]. Low bioavailability is related to low plasma and tissues concentration, rapid metabolism, and diminished effects of the active compound [23]. Other formulations of curcumin, such as nanoparticles and microparticles, are used to enhance curcumin permeability, to increase absorption and also to offer stronger resistance to rapid metabolism, and rapid systemic elimination [24,25,26,27,28]. Both curcumin solution and curcumin nanoparticles were demonstrated to have an antioxidant effect on STZ-induced DM [7,29] and also to reduce inflammation and oxidative stress in the case of ISO-induced AMI [13,14]. To the best of our knowledge, no investigation was conducted in AMI associated with DM.

Taking into consideration that efficacy of curcumine nanoparticle on DM [7,29] respectively on AMI [13,14] was already demonstrated, our hypothesis was that curcumin nanoparticles might also have a beneficial effect on AMI induced in DM patients. The present study was designed to evaluate the cardio-protective effects of pretreatment with curcumin nanoparticles compared to conventional curcumin on the changes in oxidative stress parameters and inflammatory cytokine levels during isoproterenol-induced acute myocardial infarction in rats with diabetes mellitus.

## 2. Materials and Methods 

### 2.1. Ethics Statement

The Ethics Committee of the Iuliu Hațieganu University of Medicine and Pharmacy Cluj-Napoca approved the study (Reg. No. 53/22.01.2018). Furthermore, the Sanitary-Veterinary and Food Safety Directorate from Cluj-Napoca approval (Reg. No. 99/21.02.2018) was also obtained before the beginning of the study. All the experimental procedures were carried out according to the national and international guidelines for the care and use of animals, and they followed the Helsinki Declaration on animal studies.

### 2.2. Chemicals and Drugs

Streptozotocin (STZ), curcumin (CUS), and isoproterenol hydrochloride (ISO) were purchased from Sigma-Aldrich (St. Louis, USA), and curcumin nanoparticles (CUN) were obtained from CVI Pharma (Hanoi, Vietnam). In the curcumin nanoparticles, the active compound was enclosed in polymer-based nanoparticles sized between 30 and 100 nm. Curcumin was transformed into nanosized molecules, using high-frequency ultrasonic waves. In order to keep the curcumin particles well dispersed in water and to assure an increased absorption (up to 95%), biocompatible water-based polymers were used for the curcumin nanoparticles’ preparation.

### 2.3. Animals

Fifty-six Wistar-Bratislava white male rats, weighing 200–250 g, were obtained from the Animal Department of the Faculty of Medicine, Iuliu Haţieganu University of Medicine and Pharmacy Cluj-Napoca. Rats were kept in polypropylene cages under regular 12 h:12 h day/night cycle, 40–60% relative humidity, and constant room temperature (22 ± 5 °C) in the Pathophysiology Department. Animals had free access to food and water 24 h per day. 

### 2.4. Experimental Protocol

#### 2.4.1. Groups of Study 

The animals were randomized into eight groups of seven rats/group and received pretreatment (relating DM and AMI) according to the design presented in Table 1.

#### 2.4.2. Induction of Diabetes Mellitus

Diabetes mellitus was induced on the 7th day (in DM-C, DM-AMI-C, S-DM-CUS-AMI, CUS-DM-CUS-AMI, S-DM-CUN-AMI, and CUN-DM-CUN-AMI groups) in overnight fasted rats by intraperitoneal administration of a single dose of STZ (65 mg/kg bw, bodyweight) freshly dissolved in 0.1 M of citrate buffer (pH 4.5) [30]. In the first 48 h after STZ administration, rats were provided with free access to water with 5% glucose to prevent hypoglycemic shock [30]. Forty-eight hours later, blood samples were taken from the rats’ tails, and glucose levels were measured with a glucometer (VivaChek Biotech (Hangzhou) Co., Ltd, Hangzhou, China). Rats with glucose higher or equal to 200 mg/dL were considered to have diabetes mellitus (DM) [31]. 

#### 2.4.3. Induction of Acute Myocardial Infarction

Acute myocardial infarction (AMI) was induced on the 22nd day (in AMI-C, DM-AMI-C, S-DM-CUS-AMI, CUS-DM-CUS-AMI, S-DM-CUN-AMI, and CUN-DM-CUN-AMI groups) with ISO, which was freshly dissolved in normal saline (0.09%) and injected subcutaneously in a dose of 45 mg/kg bw [11].

#### 2.4.4. Curcumin and Curcumin Nanoparticles Administration

The dose of 200 mg/kg bw of curcumin and curcumin nanoparticle were chosen based on our previously reported results, as they offer myocardial protection in AMI [13,14]. In our study, CUS and CUN were dissolved in peanut oil and were administered by gavage.

### 2.5. Biochemical Assays

On day 23, 24 h after the ISO administration, blood samples were collected from the retroorbital plexus, with rats placed under general anesthesia with xylazine (2 mg/kg, ip) and ketamine (20 mg/kg, ip). Afterward, the rats were sacrificed by an overdose of anesthetics. Serum activites of creatine kinase (CK), creatine kinase-MB (CK-MB), and lactate dehydrogenase (LDH) were measured with a Jasco V-530 UV–Vis spectrophotometer (Jasco International Co. Ltd., Tokyo, Japan), using commercial kits from Spinreact, Girona, Spain. Oxidative stress markers were assessed with a Jasco V-530 UV–Vis spectrophotometer (Jasco International Co. Ltd., Tokyo, Japan), using the methods previously described: malondialdehyde (MDA) [32], the indirect assessment of NO synthesis (NOx) [33], total oxidative status (TOS) [34], total antioxidative capacity (TAC) [35], and thiols [36]. The serum levels of thee inflammatory cytokines, tumor necrosis factor-alpha (TNF-α), interleukin-6 (IL-6), and interleukin-1β (IL-1β), were measured using the ELISA technique (Stat Fax 303 Plus Microstrip Reader, Minneapolis, USA), with commercially available kits (rat TNF-α, IL-6 and IL-1β ABTS ELISA Development kits, PeproTech EC, Ltd., London, UK).

### 2.6. Statistical Analysis

Statistical analyses were performed with Statistica 13 software (v. 13, StatSoft, USA). Our obtained data were expressed as mean and standard deviation. The distribution of investigated biochemical parameters in groups was plotted as individual values (circles) and median (line), as recommended by Weissgerber and coauthors [37]. The Mann–Whitney test was used to assess the differences between groups. *P* < 0.05 was considered as a level of significance.

## 3. Results

Statistical analysis was performed on all seven rats in each group, as no rat died during the follow-up. All rats from DM groups were definitely diabetic, proved by glycaemia >200 mg/dL, 48 h after STZ administration (as presented in Table 2). AMI was successfully induced to AMI groups after ISO administration, proved by the elevation of CK, CK-MB, and LDH (Table 3). 

### 3.1. Serum Levels of Myocardial Infarction Enzymes

**Figure 1:**
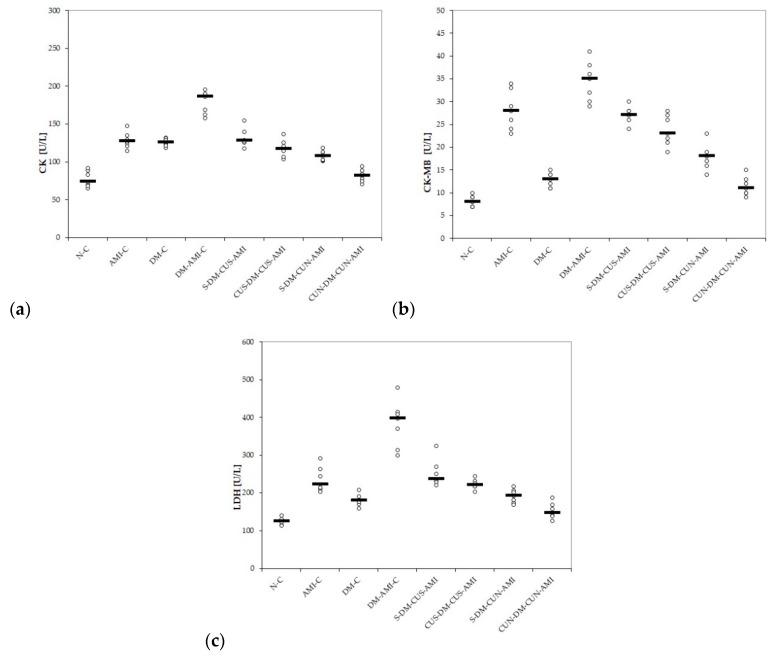
Variation of serum levels of myocardial infarction enzymes by groups: (**a**) CK (creatine kinase), (**b**) CK-MB (creatine kinase-MB), and (**c**) LDH lactate dehydrogenase by groups. Notes: The circles represent the individual values, and the horizontal line is given by the median. Abbreviations: N = normal; C = control; AMI = acute myocardial infarction; DM = diabetes mellitus; S = saline; CUS = curcumin solution in a dose of 200 mg/kg bw; CUN200 = curcumin nanoparticles solution in a dose of 200 mg/kg bw.

Administration of ISO led to increased serum levels of CK, CK-MB, and LDH (Table 3 and Figure 1a–c). Diabetic rats also presented an elevation of CK, CK-MB, and LDH, after STZ administration, with the highest levels being in those with DM and AMI (Table 3 and Figure 1a–c). The *p*-values comparing the myocardial infarction enzymes between different groups are presented in Table 4.

Both groups of rats that received CUS had lower levels of CK compared to the DM-AMI-C group (*p* = 0.0022), with no differences compared to the AMI-C group or DM-C group (*p* > 0.05, Figure 1a). Better results were obtained for the groups treated with CUN (*p* ≤ 0.0033, Table 3 and Figure 1a). All rats treated with ISO presented elevated levels of CK-MB and LDH (Table 3 and Figure 1b,c). Diabetic rats from the DM-C group also presented a slight elevation of CK-MB and LDH (Table 3 and Figure 1b,c). CUS and CUN prevented the elevation of these enzymes in all the treated groups compared to the DM-AMI-C group (*p* ≤ 0.0049, Figure 1b,c). No differences were found between the two groups that received CUS, or when they were compared to AMI-C group (*p* > 0.05, Figure 1b,c). Best results in prevention of CK-MB and LDH elevation were obtained for the groups that received CUN (*p* ≤ 0.022, Table 3 and Figure 1b,c).

### 3.2. Serum Levels of Oxidative Stress Parameters

**Figure 2:**
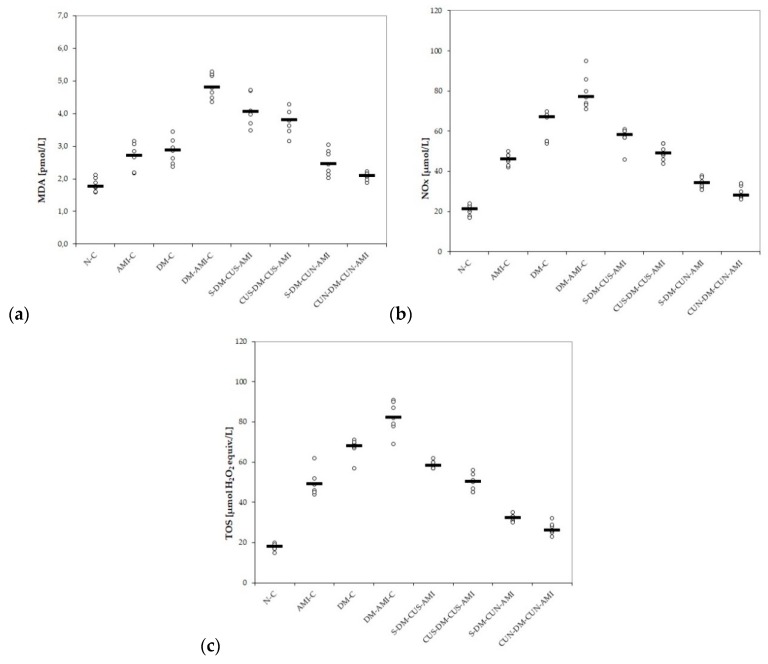
Variation by groups of serum oxidative stress intensity: (**a**) MDA (malondialdehyde), (**b**) NOx (nitric oxide), and (**c**) TOS (total oxidative status) by groups. Notes: The circles represent the individual values, and the horizontal line is given by the median. Abbreviations: N = normal; C = control; AMI = acute myocardial infarction; DM = diabetes mellitus; S = saline; CUS = curcumin solution in a dose of 200 mg/kg bw; CUN200 = curcumin nanoparticles solution in a dose of 200 mg/kg bw.

Our results showed increased serum levels of MDA, NOx, and TOS in rats with AMI and also in those with DM, with the highest level being in rats with DM, and associated AMI (Table 5 and Figure 2a–c) with significantly better results on CUS and CUN compared with controls, and CUN compared with CUS (Table 6).

CUS and CUN administration prevented elevation of MDA, NOx, and TOS compared to the DM-AMI-C group, with the best results obtained for CUN administered before DM induction (*p* ≤ 0.0407, Table 5 and Figure 2a–c). 

No differences were found between the two groups treated with CUS for MDA (*p* ≥ 0.05, Table 5 and Figure 2a), while, for NOx and TOS, lower levels were found in the group that received CUS before STZ administration (*p* ≤ 0.0176, Table 5 and Figure 2b,c).

**Figure 3:**
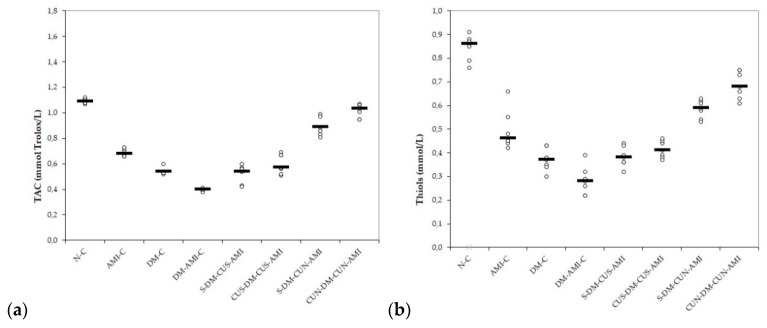
Variation by groups of serum antioxidant capacity: (**a**) TAC (total antioxidant capacity) and (**b**) thiols by groups. Notes: The circles represent the individual values, and the horizontal line is given by the median. Abbreviations: N = normal; C = control; AMI = acute myocardial infarction; DM = diabetes mellitus; S = saline; CUS = curcumin solution in a dose of 200 mg/kg bw; CUN200 = curcumin nanoparticles solution in a dose of 200 mg/kg bw.

According to our results, not only AMI but also DM induction led to a reduction in bot antioxidative markers: TAC and thiols, with lower levels in diabetic rats with AMI (Table 7 and Figure 3a,b). CUS administration before STZ administration did not offer improved results (*p* ≥ 0.05, Table 7 and Figure 3a,b), while CUN pretreatment administration before DZ induction offered better results for TAC and Thiols (*p* ≤ 0.0104, Table 7 and Figure 3a,b).

### 3.3. Serum Levels of Pro-Inflammatory Cytokines

**Figure 4:**
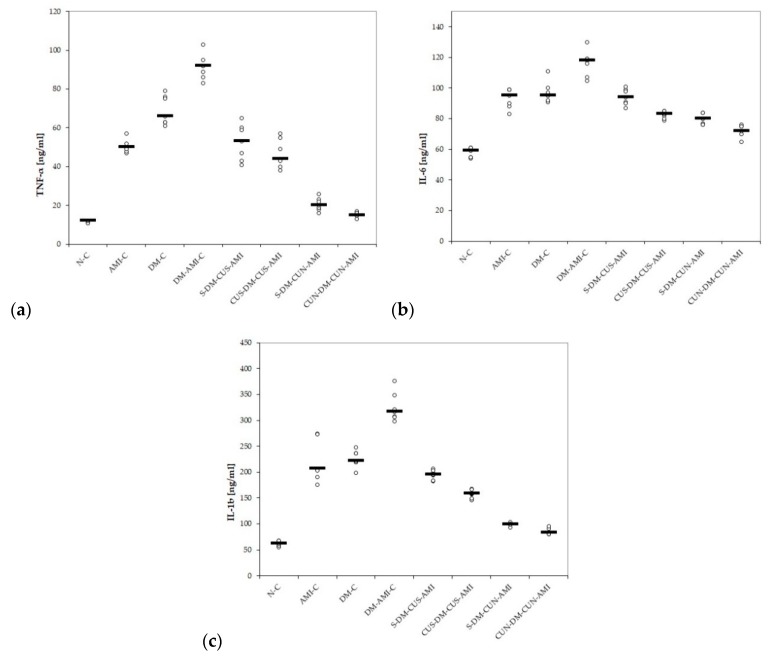
Variation by groups of serum levels of pro-inflammatory cytokines: (**a**) TNF-α (tumor necrosis factor-alpha), (**b**) IL-6 (interleukin 6), and (**c**) IL-1β (interleukin 1β), by groups. Notes: The circles represent the individual values, and the horizontal line is given by the median. Abbreviations: N = normal; C = control; AMI = acute myocardial infarction; DM = diabetes mellitus; S = saline; CUS = curcumin solution in a dose of 200 mg/kg bw; CUN200 = curcumin nanoparticles solution in a dose of 200 mg/kg bw.

The serum levels of TNF-α, IL-6, and IL-1β were increased in all the study groups, but the most elevated values were in those rats with DM and associated AMI (Table 8 and Figure 4a–c). CUS and CUN administration proved to prevent elevation of all studied cytokines compared to the group with DM and AMI, with significantly better effect for CUN administered from day one (*p* ≤ 0.0046, Table 8 and Figure 4a–c). CUS administered before STZ administration didn’t prevent the elevation of TNF-α in diabetic rats with AMI, compared to the group pretreated with saline; however, it had better results for IL-6 and IL-1β (*p* ≤ 0.0022, Table 8 and Figure 4a–c). The comparison of groups regarding the pro-inflammatory cytokines is presented in Table 9.

## 4. Discussion

### 4.1. Myocardial Infarction Enzymes

In our study, STZ and ISO administration led to elevated levels of CK, CK-MB, and LDH, with the highest levels being in rats from the DM-AMI-C group. CUS and CUN administration prevented elevation of these enzymes compared to DM-AMI-C group, with the best results obtained for those that received CUN (Table 3, Table 4, and Figure 1b,c). ISO is known to exaggerate myocardial oxygen consumption by enhancing both heart rate and contractility through activation of b-adrenoceptors [12]. These will lead to a perturbation of the physiological oxidant/antioxidant balance, to increased lipid peroxidation and to an antioxidant enzymes depletion [12]. ISO administration and hyperglycaemia from DM might increase reactive oxygen species (ROS) production. Excessive production of ROS is a key process involved in promoting myocardial damage during AMI [6]. Once myocardium is damaged, CK, CK-MB, LDH, aspartate aminotransferase (AST), and cardiac troponin I and C are released into the bloodstream, and their measurement is used as a biochemical indicator of myocardial injury [38]. LDH is expressed in many organs, including the heart, skeletal muscle, liver, kidneys, lungs, and erythrocytes, so it is not highly specific to the heart [39]. Its serum level increases within 6–12 h from the onset of AMI, has a peak over 1–3 days, and usually returns to the normal values after 8–14 days [40]. CK-MB is found not only in the heart but also in tongue, small intestine, diaphragm, uterus, prostate, and skeletal muscle [41]. Sensitivity and specificity of CK-MB in the diagnosis of AMI is given by the fact that about 20% of total CK in the myocardium tissue is an MB form, but an increased level during inflammation or trauma reduces its specificity [37]. Both levels of CK and CK-MB start to increase 4–9 h after myocardial injury, reach their highest point within 24 h, and decrease to the normal range after 48–72 h [42]. Total CK and CK-MB levels are important prognosis predictors, since they are correlated with infarct size [40,43]. A limitation of CK-MB usage is that it cannot detect minor myocardial damage, but one major advantage of CK-MB over the cardiac troponin I and T is the early clearance that helps the detection of reinfarction [44].

It was previously reported that curcumin preserves the normal structure of the cardiomyocites, increases their vitality, and minimizes elevation of the plasma cardiac enzyme markers CK, CK–MB, and LDH, following myocardial injury [45,46]. The better cardio-protective effect offered by curcumin nanoparticles can be explained by the increased bioavailability of curcumin nanoparticles, which eventually improves the delivery of the drug to the infarcted area [47].

### 4.2. Oxidative Stress Parameters

To our best knowledge, this is the first study that evaluates the antioxidative effects of curcumin nanoparticles compared to conventional curcumin on ISO-induced AMI in diabetic rats. Our results demonstrate that pretreatments with CUS and CUN have antioxidative effects on ISO-induced AMI in diabetic rats. CUS and CUN prevented the elevation in MDA, TOS, and NOx (Table 5, Table 6, and Figure 2a–c) and increased antioxidative markers, such as TAC and thiols. CUN performed better in preventing the elevation of the studied pro-oxidant parameters and in increasing antioxidant markers when it was administered before DM induction (Table 5 and Figure 2a–c). Oxidative stress has an important role in the pathogenesis of diabetes and its complications, as recent data have shown that STZ produces an imbalance between plasma oxidant and antioxidant content, resulting in diabetes [48]. MDA serves as an important indicator of tissue oxidative stress, being a degradation product of lipid peroxidation [49]. Oxidative stress in DM is caused by hyperglycemia correlated with increased free-radical formation, resulting in increased lipid peroxidation [49,50]. In AMI, MDA may accumulate due to the low oxygen level and oxidative stress induced by acute ischemic injury [51]. MDA reacts with the cellular membrane proteins changing its antigenic proprieties and producing a dysfunctional activity with consequences on cells integrity [52]. Serum levels of the inorganic nitrites and nitrates (NOx), stable end metabolites of nitric oxide (NO), were determined to evaluate the NO production, another biomarker of nitro-oxidative stress [53]. In DM, hyperglycemia and oxidative stress activate the nuclear factor NF-κB (NF-κB) pathway and increase the expression of the inducible isoform of the nitric oxide synthase (iNOS) gene, resulting in an increased production of nitric oxide NO [54]. Elevated NO levels initiate the cascade of the apoptosis pathway, impairing normal tissue functions and structure [54]. Even more, NO overproduction may act as a pro-inflammatory molecule, after conversion to peroxynitrite radical [55]. In AMI, peroxynitrite radicals will exacerbate oxidative stress and myocardial apoptosis, leading to an extension of the myocardial infarction area [56]. TOS is another indicator of the degree of oxidative stress, and, in diabetic animals, TOS levels are augmented as a result of hyperglycemia and oxidative-stress induction [57]. TOS was also reported to be increased in patients with chronic ischemic heart failure [58]. In AMI, the serum level of TOS is correlated with the intensity and complexity of coronary artery disease [59]. It was suggested that TAC may represent an indicator of the protective effects of antioxidant agents, and it is used as a new biomarker for the prevention, diagnosis, and prognosis of DM and several other diseases [60]. TAC was reported to be low in patients with AMI; therefore, the antioxidant therapy may have beneficial effects in the prevention of coronary artery disease [61] Thiols are an important antioxidant system since they are able to mediate redox-signaling processes as a response to oxidative stress, playing a significant role in mitigating the lipid peroxidative effects of ROS, along with other antioxidants in the body [62,63]. Chronic inflammation associated with hyperglycemia in DM will activate the NF-κB pathway, with a consequent increase of oxidative stress and release of pro-inflammatory cytokines, resulting in an over-oxidation of thiols [64]. In patients with AMI, the increased generation of ROS, secondary to ischemia and reperfusion, will increase thiols consumption, resulting in decreased total thiols levels [65]. 

Curcumin administration could significantly decrease MDA due to its ability to reduce the hydrogen peroxide (H_2_O_2_) induced lipid peroxidation [66,67] and inhibit nitric oxide synthase activity by reducing NO production [68]. Even more, curcumin was observed to have a modulatory role in oxidative stress [69], so pretreatment with curcumin can lead to a decreased level of TOS. Regarding the antioxidative capacity, curcumin may act by inducing a reduction in ROS production and a secondary increase in TAC levels [70]. By induction of glutathione biosynthesis and inhibition of theNF-κB pathway, curcumin administration can increase thiols levels [71]. CUN has better-reduced the levels of MDA, NOx, and TOS and increased TAC and thiols levels, due to its administration in encapsulated nanocarriers, increaseing the antioxidant properties of the active compound [72]. Even more, it was observed that empty nanoparticles in a liposomal structure might inhibit oxidative-stress-induced cell death and morphological changes that occur in the nucleus, cytoplasm, and mitochondria of cells [73].

### 4.3. Pro-Inflammatory Cytokines

In our study, CUS and CUN ensured a diminished level of inflammatory cytokines, such as TNF-α, IL-6a, and IL-1β, in ISO-induced AMI in rats with previous STZ-induced DM (Table 8 and Figure 4). CUN performed better compared to CUS in preventing the increase in the levels of cytokines mentioned above (Table 8, Table 9, and Figure 4a–c). Nowadays, DM is considered to be a low-grade chronic inflammatory condition characterized by the over-secretion of pro-inflammatory cytokines [74]. TNF-α is a potent inflammatory cytokine, released from macrophages and T lymphocytes, with important functions on DM, since it not only plays an important role in the development of insulin resistance, but also in progression to microvascular complications of DM [75]. IL-6 is another inflammatory cytokine produced in several immune cell types, mainly in endothelial cells, smooth and skeletal cells, adipocytes, and islet β-cells [76]. It also contributes to the development of both types of DM and has an influence on metabolism and glucose balance [75]. TNF-α and IL-6 influence collagen formation and are therefore involved in scar formation after AMI [77,78]. Usually TNF-α is not expressed in normal cardiomyocytes, but in the case of myocardial ischemia and anoxia, cardiomyocytes and myocardial mononuclear macrophages are activated, and they will produce large amounts of TNF-α, not only in the infarcted zone of the myocardium, but also at the infarction border zone [79]. Serum levels of IL-6 are elevated after the onset of AMI, and its levels are correlated with the extension of the infarct area [80]. IL-1β, which plays a major role in a wide array of auto-inflammatory diseases, was observed to act as the key promoter of tissue and systemic inflammation in DM [66]. IL-1β is considered to play an important role in the development of cardiovascular complications of DM, especially diabetic vasculopathy, since its release from adipokines can have an impact on distant organs, including the heart or the vessels, due to increased systemic and vascular inflammation [74]. In AMI, the increased serum levels of IL-1β cause activation of the myofibroblasts involved in cardiac remodeling, resulting in an alteration of heart systolic function [80]. The reduction of the IL-1β serum level in AMI was reported to be associated with a reduced area of the affected myocardial tissue [80,81]. 

Administration of CUS and CUN was proved to be effective in preventing the serum level elevation of TNF-α, IL-6, IL-1α, IL-1β, and RANTES after ISO-induced AMI [13], but as far as we know, this is the first study focused on the effect of curcumin and curcumin nanoparticles on TNF-α, IL-6a, and IL-1β plasma levels in AMI in diabetic rats. Based on the auto-inflammatory features of DM, the pharmacological strategies to treat diabetes should not only be focused on correcting hyperglycemia, but also on targeting chronic inflammation, in order to prevent the development of metabolic and cardiovascular complications [74]. Our results show that curcumin prevented the elevation of the abovementioned cytokines, since curcumin can reduce the inflammatory responses by interfering with NF-κB activation, a critical pathway in the regulation of transcription of pro-inflammatory related genes [68]. The enhanced anti-inflammatory effect of the curcumin nanoparticles can explain the better effects obtained after CUN administration, as a result of increased bioavailability [13]. 

### 4.4. Potential Limitations

The fact that other parameters known to be altered in the presence of DM, such as the evaluation of endogenous insulin levels, which were not assessed, could be regarded as a potential limitation of the present study. Histopathological analysis of the pancreas and heart would also have been of great interest. Another limitation of our study is that we did not find a way to check if a consistent dose of curcumin nanoparticles was administered for the rats who received CUN, since the size of the nanoparticles varied between 30 and 100 nm.

## 5. Conclusions

The findings of our study demonstrate that curcumin nanoparticles have significant cardio-protective effects, as they have both antioxidative and anti-inflammatory impacts on acute myocardial infarction in diabetes mellitus. Therefore, curcumin nanoparticles could be valuable for the development of targeted preventive strategies in reducing acute myocardial damage in diabetes mellitus.

## Figures and Tables

**Table 1:** Design of the experimental study groups.

	Day	1	2	3	4	5	6	7	7 (i.p.)	8	9	10	11	12	13	14	15	16	17	18	19	20	21	22	22 (s.c.)
Group																									
N-C		saline by gavage							**CB**	saline by gavage															**SS**
AMI-C									**CB**																**ISO**
DM-C									**STZ**																**SS**
DM-AMI-C									**STZ**																**ISO**
S-DM-CUS-AMI									**STZ**	CUS by gavage															**ISO**
CUS-DM-CUS-AMI		CUS by gavage							**STZ**																**ISO**
S-DM-CUN-AMI		saline by gavage							**STZ**	CUN by gavage															**ISO**
CUN-DM-CUN-AMI		CUN by gavage							**STZ**																**ISO**

Notes: N, normal; C, control; AMI, acute myocardial infarction; DM, diabetes mellitus; SS, saline solution (0.09%); CB, citrate buffer; CUS, curcumin solution (200 mg/kg bw); CUN, curcumin nanoparticle solution (200mg/kg bw); STZ, streptozotocin in a dose of 65 mg/kg bw; ISO, isoproterenol in a dose of 45 mg/kg bw; i.p., intraperitoneal; s.c., subcutaneous.

**Table 2:** Glycaemia levels (mg/dL) for all the rats at 48 h after streptozotocin administration.

Rat No.	N-C	AMI-C	DM-C	DM-AMI-C	S-DM-CUS-AMI	CUS-DM-CUS-AMI	S-DM-CUN-AMI	CUN-DM-CUN-AMI
1	80	76	544	549	497	419	389	375
2	75	78	532	498	462	452	362	366
3	69	79	559	534	526	432	435	324
4	81	82	562	504	432	417	441	272
5	82	74	468	518	478	432	360	389
6	78	75	563	569	508	447	380	262
7	74	86	434	479	458	440	429	288

Notes: N, normal; C, control; AMI, acute myocardial infarction; DM, diabetes mellitus; S, saline; CUS, curcumin solution; CUN, curcumin nanoparticle solution; CUS200, curcumin solution in a dose of 200 mg/kg bw; CUN200, curcumin nanoparticle solution in a dose of 200 mg/kg bw.

**Table 3:** Serum levels of myocardial infarction enzymes.

Group Abbreviation	CK (U/L)	CK-MB (U/L)	LDH (U/L)
N-C	77.43 (10.6)	8.14 (1.21)	124.57 (9.48)
AMI-C	128.71 (10.63)	28.14 (4.22)	235.86 (32.43)
DM-C	126.14 (4.81)	12.86 (1.57)	180.57 (15.46)
DM-AMI-C	178.71 (15.03)	34.43 (4.35)	383.43 (61.77)
S-DM-CUS-AMI	131.86 (12.08)	27.14 (1.86)	252.00 (36.07)
CUS-DM-CUS-AMI	118.14 (11.26)	23.71 (3.35)	222.86 (13.02)
S-DM-CUN-AMI	107.86 (6.54)	17.86 (2.79)	191.71 (18.13)
CUN-DM-CUN-AMI	81.86 (7.99)	11.43 (2.07)	152.00 (20.84)

Notes: values expressed as mean (standard deviation). Abbreviations: CK, creatine kinase; CK-MB, creatine kinase-MB; LDH, lactate dehydrogenase; N, normal; C, control; AMI, acute myocardial infarction; DM, diabetes mellitus; S, saline; CUS, curcumin solution in a dose of 200 mg/kg bw; CUN200, curcumin nanoparticles solution in a dose of 200 mg/kg bw.

**Table 4:** *P*-values for comparisons between the study groups for myocardial infarction enzymes.

Group Abbreviation	CK (U/L)	CK-MB (U/L)	LDH (U/L)
S-DM-CUS-AMI vs.			
AMI-C	0.6544	0.7968	0.3067
DM-C	0.5220	0.0021	0.0022
DM-AMI-C	0.0022	0.0039	0.0049
CUS-DM-CUS-AMI vs.			
AMI-C	0.1244	0.0630	0.7491
DM-C	0.1098	0.0021	0.0033
DM-AMI-C	0.0022	0.0022	0.0022
S-DM-CUS-AMI	0.0348	0.0613	0.0409
S-DM-CUN-AMI vs.			
AMI-C	0.0033	0.0026	0.0106
DM-C	0.0026	0.0047	0.3067
DM-AMI-C	0.0022	0.0021	0.0022
S-DM-CUS-AMI	0.0033	0.0021	0.0022
CUN-DM-CUN-AMI vs.			
AMI-C	0.0022	0.0021	0.0022
DM-C	0.0022	0.1754	0.0215
DM-AMI-C	0.0022	0.0021	0.0022
CUS-DM-CUS-AMI	0.0022	0.0021	0.0022
S-DM-CUN-AMI	0.0022	0.0032	0.0073

Abbreviations: CK, creatine kinase; CK-MB, creatine kinase-MB; LDH, lactate dehydrogenase; N, normal; C, control; AMI, acute myocardial infarction; DM, diabetes mellitus; S, saline; CUS, curcumin solution in a dose of 200 mg/kg bw; CUN200, curcumin nanoparticles solution in a dose of 200 mg/kg bw.

**Table 5:** Serum levels oxidative stress intensity.

Group Abbreviation	MDA (nmol/L)	NOx (μmol/L)	TOS (μmol H_2_O_2_ equiv./L)
N-C	1.82 (0.20)	20.86 (2.67)	17.71 (1.60)
AMI-C	2.69 (0.39)	46.00 (2.89)	49.57 (6.13)
DM-C	2.84 (0.39)	62.43 (7.32)	67.14 (4.67)
DM-AMI-C	4.85 (0.37)	79.43 (8.50)	82.29 (7.78)
S-DM-CUS-AMI	4.10 (0.47)	57.00 (5.10)	58.86 (1.86)
CUS-DM-CUS-AMI	3.74 (0.37)	49.43 (3.82)	49.71 (4.31)
S-DM-CUN-AMI	2.50 (0.39)	34.29 (2.56)	32.14 (1.68)
CUN-DM-CUN-AMI	2.08 (0.11)	29.43 (3.05)	27.00 (2.94)

Notes: values expressed as mean (standard deviation). Abbreviations: MDA, malondialdehyde; NOx, nitric oxide; TOS, total oxidative status; N, normal; C, control; AMI, acute myocardial infarction; DM, diabetes mellitus; S, Saline; CUS, curcumin solution in a dose of 200 mg/kg bw; CUN200, curcumin nanoparticles solution in a dose of 200 mg/kg bw.

**Table 6:** *P*-values for comparisons between the study groups for oxidative stress parameters.

	MDA (nmol/L)	NOx (μmol/L)	TOS (μmol H_2_O_2_ equiv./L)	TAC (mmol Trolox/L)	Thiols (mmol/L)
S-DM-CUS-AMI vs.					
AMI-C	0.0022	0.0085	0.0245	0.0020	0.0059
DM-C	0.0022	0.4413	0.0205	0.7433	0.5627
DM-AMI-C	0.0215	0.0021	0.0021	0.0020	0.0147
CUS-DM-CUS-AMI vs.					
AMI-C	0.0026	0.1079	0.6999	0.0825	0.0344
DM-C	0.0049	0.0031	0.0021	0.2737	0.0727
DM-AMI-C	0.0022	0.0021	0.0021	0.0020	0.0059
S-DM-CUS-AMI	0.1792	0.0176	0.0021	0.1994	0.1400
S-DM-CUN-AMI vs.					
AMI-C	0.4433	0.0021	0.0021	0.0020	0.0553
DM-C	0.1252	0.0021	0.0021	0.0020	0.0021
DM-AMI-C	0.0022	0.0022	0.0021	0.0020	0.0021
S-DM-CUS-AMI	0.0022	0.0021	0.0020	0.0021	0.0021
CUN-DM-CUN-AMI vs.					
AMI-C	0.0049	0.0021	0.0021	0.0020	0.0059
DM-C	0.0021	0.0021	0.0021	0.0020	0.0021
DM-AMI-C	0.0021	0.0021	0.0021	0.0020	0.0021
CUS-DM-CUS-AMI	0.0021	0.0021	0.0021	0.0021	0.0021
S-DM-CUN-AMI	0.0407	0.0210	0.0085	0.0104	0.0071

Abbreviations: CK, creatine kinase; CK-MB, creatine kinase-MB; LDH, lactate dehydrogenase; N, normal; C, control; AMI, acute myocardial infarction; DM, diabetes mellitus; S, saline; CUS, curcumin solution in a dose of 200 mg/kg bw; CUN200, curcumin nanoparticles solution in a dose of 200 mg/kg bw.

**Table 7:** Serum levels of antioxidant capacity.

Group Abbreviation	TAC (mmol Trolox/L)	Thiols (mmol/L)
N-C	1.09 (0.02)	0.85 (0.05)
AMI-C	0.68 (0.03)	0.49 (0.08)
DM-C	0.54 (0.03)	0.37 (0.05)
DM-AMI-C	0.40 (0.01)	0.28 (0.06)
S-DM-CUS-AMI	0.52 (0.07)	0.38 (0.04)
CUS-DM-CUS-AMI	0.60 (0.08)	0.41 (0.04)
S-DM-CUN-AMI	0.89 (0.07)	0.59 (0.04)
CUN-DM-CUN-AMI	1.02 (0.05)	0.69 (0.06)

Notes: values expressed as mean (standard deviation). Abbreviations: TAC, total antioxidant capacity; N, normal; C, control; AMI, acute myocardial infarction; DM, diabetes mellitus; S, saline; CUS, curcumin solution in a dose of 200 mg/kg bw; CUN200, curcumin nanoparticles solution in a dose of 200 mg/kg bw.

**Table 8:** Serum levels of pro-inflammatory cytokine.

Group Abbreviation	TNF-α (pg/mL)	IL-6 (pg/mL)	IL-1β (pg/mL)
N-C	11.71 (0.49)	58.00 (3.21)	62.00 (5.00)
AMI-C	50.43 (3.31)	93.29 (6.40)	218.71 (39.98)
DM-C	69.00 (7.42)	97.29 (6.75)	225.43 (15.99)
DM-AMI-C	91.43 (6.50)	116.29 (8.36)	325.00 (27.80)
S-DM-CUS-AMI	52.57 (9.20)	94.29 (5.22)	194.86 (9.26)
CUS-DM-CUS-AMI	46.57 (7.32)	82.43 (2.30)	157.86 (8.11)
S-DM-CUN-AMI	20.57 (3.36)	79.57 (3.46)	99.29 (3.30)
CUN-DM-CUN-AMI	15.29 (1.25)	72.14 (3.80)	85.14 (5.64)

Notes: values expressed as mean (standard deviation), Abbreviations: TNF-α, tumor necrosis factor-alpha; IL-6, interleukin 6; IL-1β, interleukin 1β; N, normal; C, control; AMI, acute myocardial infarction; DM, diabetes mellitus; S, saline; CUS, curcumin solution in a dose of 200 mg/kg bw; CUN200, curcumin nanoparticles solution in a dose of 200 mg/kg bw.

**Table 9:** *P*-values for comparisons between the study groups for pro-inflammatory cytokine.

	TNF-α (pg/mL)	IL-6 (pg/mL)	IL-1β (pg/mL)
S-DM-CUS-AMI vs.			
AMI-C	0.7489	0.8971	0.2222
DM-C	0.0072	0.4812	0.0058
DM-AMI-C	0.0021	0.0021	0.0022
CUS-DM-CUS-AMI vs.			
AMI-C	0.2486	0.0067	0.0021
DM-C	0.0021	0.0021	0.0021
DM-AMI-C	0.0021	0.0021	0.0022
S-DM-CUS-AMI	0.2243	0.0021	0.0022
S-DM-CUN-AMI vs.			
AMI-C	0.0021	0.0046	0.0020
DM-C	0.0021	0.0021	0.0020
DM-AMI-C	0.0021	0.0021	0.0021
S-DM-CUS-AMI	0.0022	0.0021	0.0021
CUN-DM-CUN-AMI vs.			
AMI-C	0.0020	0.0020	0.0021
DM-C	0.0020	0.0021	0.0021
DM-AMI-C	0.0020	0.0021	0.0022
CUS-DM-CUS-AMI	0.0020	0.0021	0.0022
S-DM-CUN-AMI	0.0046	0.0030	0.0032

Abbreviations: CK, creatine kinase; CK-MB, creatine kinase-MB; LDH, lactate dehydrogenase; N, normal; C, control; AMI, acute myocardial infarction; DM, diabetes mellitus; S, saline; CUS, curcumin solution in a dose of 200 mg/kg bw; CUN200, curcumin nanoparticles solution in a dose of 200 mg/kg bw.

## Data Availability

The data obtained from the experiment can be obtained upon reasonable request addressed to Paul-Mihai Boarescu (e-mail: boarescu.paul@umfcluj.ro) and will be publicly available after publication of the associated PhD thesis.
